# Clinical value of wide-angle colonoscopy combined with narrow-band imaging in detecting sessile serrated lesions during colorectal cancer screening: A single-center retrospective observational study

**DOI:** 10.1097/MD.0000000000049637

**Published:** 2026-07-10

**Authors:** Bin Liu, Haiyan Wang, Lin Li, Xia Huang, Liangting Hou

**Affiliations:** a Department of Proctology, Chongqing Rongchang Hospital of Traditional Chinese Medicine, Chongqing, China; b Department of Spleen and Stomach Diseases, Chongqing Rongchang Hospital of Traditional Chinese Medicine, Chongqing, China.

**Keywords:** colorectal cancer screening, detection rate, narrow-band imaging, sessile serrated lesion, wide-angle colonoscopy

## Abstract

Sessile serrated lesions (SSLs) represent a clinically significant challenge in colorectal cancer screening due to their flat morphology and association with interval cancers. Advanced endoscopic techniques such as wide-angle colonoscopy (WAC) and narrow-band imaging (NBI) may enhance detection rates of SLs, but evidence regarding their combined efficacy remains limited. This study aimed to evaluate the effectiveness of WAC combined with NBI in detecting SSLs compared to NBI alone and standard white-light endoscopy (WLE) during colorectal cancer (CRC) screening. In this single-center retrospective observational cohort study, the clinical records of 342 eligible patients who underwent CRC screening at Rongchang District Hospital between January 4, 2024, and January 31, 2025, were reviewed. Data were extracted from the Epic-Hyperspace electronic medical record system and institutional endoscopy/pathology records. Patients were categorized according to the imaging strategy documented during colonoscopy: WAC + NBI, full procedure with 170° view and NBI, n = 114; WLE + NBI, white-light examination with selective NBI activation, n = 114; or WLE + WAC, 170° wide-angle without NBI, n = 114. The primary outcome was sessile serrated lesion detection rate. Endoscopic procedures were performed using Olympus 290 systems. Histopathological diagnosis was conducted by pathologists blinded to the imaging group. In unadjusted comparisons, the WAC + NBI group had a higher sessile serrated lesion detection rate (18.4%) than both the WLE + NBI group (11.4%, *P* < .05, and the WLE + WAC group (7.9%, *P* < .05. No significant differences were observed in most secondary outcomes. The exploratory adenoma miss rate in a 10% subsample was numerically lower in WAC + NBI (11.1%) than in WLE + WAC (27.8%, *P* > .05. Procedurally, WAC + NBI required longer withdrawal times (9.3 ± 1.5 minutes) than comparator groups (8.0–8.1 minutes, *P* < .05. The combination of WAC and NBI may offer clinical advantages in improving SSL detection during CRC screening. These findings could support broader adoption of advanced endoscopic technologies in population-based screening programs. These findings should be interpreted cautiously and validated in prospective multicenter studies.

## 1. Introduction

Colorectal cancer (CRC) remains a significant global health burden, ranking among the leading causes of cancer-related morbidity and mortality worldwide.^[1]^ While conventional adenomas have long been considered the primary precursors to CRC, increasing evidence indicates that serrated lesions (SLs), particularly sessile serrated lesions (SSLs), play a substantial role in the serrated neoplasia pathway of colorectal carcinogenesis.^[2–4]^

SSLs progress to CRC through a distinct serrated pathway.^[5]^ They are typically characterized by a flat or slightly elevated morphology, indistinct borders, an overlying mucous cap, and a predilection for the proximal colon, making them difficult to detect with standard white-light endoscopy (WLE).^[6,7]^ Studies indicate that the miss rate for SSLs significantly exceeds that of conventional adenomas under WLE, potentially accounting for a substantial proportion of interval CRC.^[8,9]^ Furthermore, SSLs rarely bleed, reducing the sensitivity of noninvasive screening tests such as fecal occult blood tests.^[10]^ Differentiating SSLs from hyperplastic polyps remains a diagnostic challenge, both endoscopically and histologically, resulting in inconsistencies in detection and diagnosis.^[11,12]^ Consequently, enhancing the detection of SSLs is paramount for optimizing the effectiveness of CRC screening programs and reducing interval cancer rates.

Colonoscopy is considered the gold standard for CRC screening, and proper identification and management of colorectal polyps is an important quality measure for colonoscopy results.^[13,14]^ Wide-angle colonoscopy (WAC) systems provide an expanded field of view, potentially minimizing mucosal blind spots and improving the initial visualization rate of lesions, particularly those located behind folds or at flexures.^[15]^ Likewise, narrow-band imaging (NBI), an image-enhanced endoscopy technique, utilizes filtered light to emphasize mucosal surface patterns and microvascular architecture, facilitating the detection of subtle lesions.^[16]^ Despite these individual technological improvements, robust evidence regarding the combined efficacy of WAC and NBI for SSL detection remains limited and inconclusive.^[17–20]^ An expanded field of view alone may be insufficient to discern the subtle surface features diagnostic of SSLs, while reliance solely on image enhancement might still miss lesions outside a potentially narrower effective field.^[21–23]^

This study aimed to compare the detection performance associated with WAC combined with NBI versus NBI alone and WAC alone, each combined with standard white-light endoscopy, in the detection of SSLs during colorectal cancer screening. We hypothesized that the combined use of WAC and NBI would be associated with a higher SSL detection rate than either modality used without the other.

## 2. Materials and methods

### 2.1. Study design and ethical issues

This single-center retrospective observational cohort study was approved by the Ethics Committee of Chongqing Rongchang District Hospital of Traditional Chinese Medicine and was conducted in accordance with the Declaration of Helsinki. The analysis used de-identified data extracted from the Epic-Hyperspace electronic medical record system and institutional endoscopy and pathology records. Given the retrospective nature of the study, the requirement for individual written informed consent was waived by the ethics committee. No prospective randomization or intervention allocation was performed.

### 2.2. Setting and participants

Participants were recruited from patients undergoing colorectal cancer screening at Rongchang District Hospital between January4, 2024 and January 31, 2025. Inclusion criteria were: age ≥40 years; indication for colonoscopy screening and undergoing screening or diagnostic colonoscopy; and availability of complete endoscopy and pathology records. Exclusion criteria included: history of severe cardiopulmonary, hepatic, or renal disease; pregnancy or lactation; suspected or confirmed bowel obstruction, stricture, or perforation; history of familial polyposis syndromes; and contraindications for colonoscopy as assessed by the investigator.

### 2.3. Group categorization

The 342 eligible patients were categorized into 3 groups according to the imaging strategy documented in the endoscopy record: WAC + NBI group, n = 114, entire procedure with 170° wide-angle view and NBI; WLE + NBI group, n = 114, white-light examination with selective NBI activation for suspicious lesions; and WLE + WAC group, n = 114, white-light examination with 170° wide-angle view and NBI disabled. Endoscopist experience, experienced versus less experienced, was extracted from the record and considered when interpreting group comparability. Because group membership reflected clinical practice rather than random allocation, potential confounding by operator preference, withdrawal time, lesion suspicion, and patient characteristics could not be completely eliminated.

### 2.4. Endoscopic procedure

All colonoscopies were performed using the CF-HQ290ZI high-definition system (CLV-290, Olympus), equipped with 170° wide-angle optics and NBI capability. The patients underwent colonoscopies while under conscious sedation with midazolam. The target colonoscope withdrawal time was 6 minutes, following the good colonoscopy practice guidelines.^[24]^ All detected lesions underwent endoscopic resection with histopathological submission.

Bowel preparation followed standard institutional protocols, and its quality was assessed using the Boston bowel preparation scale (BBPS).^[25]^ A score of 8 to 9 was considered excellent, 6 to 7 good, and <6 inadequate. All colonoscopies were performed by 4 endoscopists – 3 with >500 prior procedures (experienced) and 1 with <200 (less experienced). Withdrawal time, cecal intubation rate, and total procedure duration were recorded.

### 2.5. Histopathology

All resected lesions were fixed in 10% formalin and assessed by 2 gastrointestinal pathologists blinded to group allocation. Histopathological diagnosis followed the World Health Organization Classification of Digestive System Tumours (5th edition).^[26]^ SSLs were diagnosed based on the presence of distorted crypt architecture with a characteristic serrated pattern.^[27–29]^ Prior to the study, both pathologists underwent calibration sessions to standardize diagnostic interpretation.

### 2.6. Outcome measures

The primary outcome was sessile serrated lesion detection rate (SDR), defined as the proportion of patients with at least 1 histologically confirmed SSL. Secondary outcomes included: polyp detection rate (PDR, proportion with ≥1 polyp), adenoma detection rate (ADR, proportion with ≥1 adenoma), advanced adenoma detection rate (adenoma ≥10 mm, with villous component or high-grade dysplasia), and detection rates of diminutive polyps (≤5 mm) and flat lesions (Paris classification IIa/IIb). Additionally, adenoma miss rate was calculated in a 10% random subsample using a 2nd-pass withdrawal technique (performed 2 hours later by a blinded endoscopist).

### 2.7. Data collection and statistical analysis

Because this was a retrospective study using available eligible records from the study period, no formal prospective sample-size calculation was conducted. Demographic variables, including age, gender, and body mass index; clinical risk factors, including smoking history and family history of CRC; and endoscopic quality metrics, including BBPS scores, cecal intubation time, and withdrawal time, were extracted from the Epic-Hyperspace electronic medical record system. Fecal occult blood test, results within 3 months prior to colonoscopy were recorded when available. All variables were entered into a standardized case report form for subsequent statistical analysis.

Statistical analysis was performed using SPSS v27.0 (IBM Corp., Armonk). Continuous variables were compared among groups using 1-way analysis of variance or the Kruskal–Wallis test, as appropriate. Categorical variables were analyzed using chi-square or Fisher exact tests. Pairwise comparisons were interpreted as exploratory, and all results were considered associative rather than causal. Two-sided *P* values <.05 were considered statistically significant.

## 3. Results

### 3.1. Baseline characteristics

A total of 342 eligible patients were included and categorized into 3 groups: WAC + NBI, n = 114; WLE + NBI, n = 114; and WLE + WAC, n = 114. The baseline demographic and clinical characteristics were comparable among groups. The mean age was 59.27 ± 8.66 years, and 54.4% of participants were male. No significant differences were found in body mass index, smoking history, family history of CRC, or fecal occult blood test results, all *P* > .05 (Table 1).

**Table 1:** Baseline characteristics of study participants.

	WAC + NBI (n = 114)	WLE + NBI (n = 114)	WLE + WAC (n = 114)	*P* value
Age (yr), mean ± SD	59.1 ± 8.7	59.4 ± 8.5	59.2 ± 8.6	.940
Male, n (%)	62 (54.4%)	60 (52.6%)	64 (56.1%)	.588
BMI (kg/m^2^), mean ± SD	24.5 ± 3.1	24.6 ± 3.0	25.31 ± 3.7	.477
Smoking history, n (%)	30 (26.3%)	32 (28.1%)	29 (25.4%)	.191
Family history of CRC, n (%)	13 (11.4%)	15 (13.2%)	14 (12.3%)	.693
Positive FOBT, n (%)	21 (18.4%)	24 (21.1%)	22 (19.3%)	.286

BMI = body mass index, CRC = colorectal cancer, NBI = narrow-band imaging, FOBT = fecal occult blood test, SD = standard deviation, WAC = wide-angle colonoscopy, WLE = white-light endoscopy.

### 3.2. Colonoscopy quality metrics

Colonoscopy quality indicators are presented in Table 2. All groups achieved near-perfect cecal intubation rates (100% for WAC + NBI and WLE + NBI, 99.1% for WLE + WAC). There were no significant differences among the 3 groups in terms of BBPS scores, cecal intubation rate, or total procedure time. However, the WAC + NBI group had a significantly longer mean withdrawal time than the other groups (*P* < .05).

**Table 2:** Colonoscopy quality indicators.

	WAC + NBI (n = 114)	WLE + NBI (n = 114)	WLE + WAC (n = 114)	*P* value
BBPS score, mean ± SD	7.4 ± 1.1	7.2 ± 1.3	7.1 ± 1.2	.164
Adequate prep (BBPS ≥ 6), n (%)	109 (95.6%)	107 (93.9%)	105 (92.1%)	.502
Withdrawal time (min), mean ± SD	9.3 ± 1.5	8.0 ± 1.4	8.1 ± 1.3	.009
Total procedure time (min), mean ± SD	21.3 ± 4.2	20.8 ± 3.9	19.9 ± 4.5	.110
Cecal intubation rate n (%)	114 (100%)	114 (100%)	113 (99.1%)	.936
Cecal intubation time (min), mean ± SD	5.1 ± 1.8	5.3 ± 2.0	5.0 ± 1.7	.441
Sedation dose, mean ± SD	3.2 ± 0.8	3.3 ± 0.7	3.1 ± 0.9	.289

BBPS = Boston bowel preparation scale, NBI = narrow-band imaging, SD = standard deviation, WAC = wide-angle colonoscopy, WLE = white-light endoscopy.

Colonoscopy quality indicators were comparable across groups (Table 2). Mean withdrawal times exceeded the 6-minute minimum standard in all groups, with WAC + NBI demonstrating the longest withdrawal time (9.3 ± 1.5 minutes). No significant differences in sedation requirements or bowel preparation adequacy were observed.

### 3.3. Detection outcomes

As shown in Table 3, the primary outcome SDR was significantly higher in the WAC + NBI group (18.4%) compared with the WLE + NBI group (11.4%) and the WLE + WAC group (7.9%) (*P* < .05). The number of SSLs per patient was also highest in the WAC + NBI group (0.21 ± 0.41 vs 0.09 ± 0.29 in WLE + WAC; *P* < .05). PDR and ADR were also highest in the WAC + NBI group, although differences were not statistically significant (*P* > .05). Detection rates of advanced adenomas, flat lesions, and diminutive polyps showed no significant group differences, though numerical trends consistently favored the WAC + NBI group.

**Table 3:** Comparison of sessile serrated lesion and other polyp detection rates among the 3 study groups.

	WAC + NBI (n = 114)	WLE + NBI (n = 114)	WLE + WAC (n = 114)	*P* value
SSL per patient, mean ± SD	0.21 ± 0.41	0.13 ± 0.34	0.09 ± 0.29	.030
SDR, n (%)	21 (18.4%)	13 (11.4%)	9 (7.9%)	.036
PDR, n (%)	54 (47.4%)	50 (43.9%)	48 (42.1%)	.672
ADR, n (%)	40 (35.1%)	36 (31.6%)	33 (29.0%)	.115
Advanced adenoma detection, n (%)	10 (8.8%)	8 (7.0%)	6 (5.3%)	.474
Diminutive polyps (≤5 mm), n (%)	46 (40.4%)	42 (36.8%)	39 (34.2%)	.291
Flat lesions detected, n (%)	17 (14.9%)	14 (12.3%)	11 (9.6%)	.386

ADR = adenoma detection rate, NBI = narrow-band imaging, PDR = polyp detection rate, SDR = sessile serrated lesion detection rate, SSL = sessile serrated lesion, WAC = wide-angle colonoscopy, WLE = white-light endoscopy..

### 3.4. Adenoma miss rate in subsample

In a randomly selected 10% subsample (n = 34), each group contributed 11 to 12 patients who underwent a 2nd-pass withdrawal. The adenoma miss rate was lowest in the WAC + NBI group (11.1%) compared with 22.2% in the WLE + NBI group and 27.8% in the WLE + WAC group, though these differences did not reach statistical significance (*P* < .05).

## 4. Discussion

In this retrospective observational cohort study, we compared SSL detection outcomes among patients examined with WAC + NBI, WLE + NBI, and WLE + WAC during colorectal cancer screening. The WAC + NBI group had a higher SSL SDR and a higher number of SSLs per patient than the WLE + NBI and WLE + WAC groups. Although the differences in PDR, ADR, and other lesion subtypes were not statistically significant, numerical trends favored the WAC + NBI group, suggesting a potential association between combined advanced imaging and SSL detection.

Hyperplastic polyps are usually considered non-neoplastic; however, SSLs are precursors of cancer via the “serrated tumor pathway,” and they are difficult to detect and completely resect.^[4,6]^ Recent data suggest that techniques such as high-definition endoscopy, chromoendoscopy, narrow-band imaging, and other image-enhanced approaches can improve lesion characterization and may improve detection rates.^[30,31]^ In the present retrospective analysis, colonoscopy using a combined 170° wide-angle view and narrow-band imaging (WAC + NBI) was associated with a higher SSL detection rate than the comparator techniques. The observed SSL detection rate of 18.4% in the WAC + NBI group represented a 61.4% relative increase over selective narrow-band imaging alone (WLE + NBI: 11.4%) and a 133% increase over wide-angle imaging without optical enhancement (WLE + WAC: 7.9%). These findings are consistent with evidence emphasizing the complementary roles of expanded mucosal visualization and vascular contrast enhancement in detecting subtle serrated lesions.^[32]^ Similarly, the use of WAC has been reported to improve adenoma detection by providing a broader field of view and reducing blind spots behind haustral folds.^[32]^

The higher SDR observed in the WAC + NBI group may stem from 2 complementary mechanisms. NBI improves contrast between neoplastic and non-neoplastic tissue by narrowing the wavelength spectrum, while WAC allows visualization of more mucosal surface at any given time.^[16,33–35]^ This dual approach may be particularly useful in detecting SSLs, which often present as flat, pale, mucus-covered lesions with indistinct margins – features that challenge both novice and experienced endoscopists.^[7]^ Additionally, the longer mean withdrawal time in the WAC + NBI group may have contributed to higher detection, although all groups exceeded the recommended 6-minute minimum threshold for withdrawal.

Interestingly, while SSL detection was higher in the WAC + NBI group, ADR and advanced adenoma detection did not differ significantly among groups. This may be due to the inherently more conspicuous appearance of conventional adenomas, making them easier to detect even with standard WLE.^[36]^ This contrasts with studies reporting NBI-driven ADR improvements.^[37]^ The adenoma miss rate in our 10% subsample also numerically favored the WAC + NBI group, though statistical significance was not achieved, likely due to small sample size and limited power in this subgroup. This observation is consistent with studies correlating WAC with reduced missed lesion rates,^[38]^ particularly for diminutive flat adenomas.

Several methodological considerations are important when interpreting these findings. Our SSL detection rate exceeded historical benchmarks of 5% to 10% under standard colonoscopy, which may relate to the use of advanced imaging, adequate withdrawal times, and blinded histopathological verification using World Health Organization diagnostic criteria. However, because data were retrospectively extracted from clinical records and groups were defined by the technique documented in routine practice, the observed differences should be interpreted as associations rather than proof of a causal effect of WAC + NBI.

This study has several limitations. First, it was a single-center retrospective observational study, which may limit generalizability and is inherently susceptible to selection bias and residual confounding. Second, no prospective randomization was performed; therefore, differences in patient characteristics, endoscopist preference, withdrawal time, lesion suspicion, and procedural context may have influenced the observed SDR. Third, although the gastrointestinal pathologists were blinded to the imaging group, endoscopists could not be blinded to the modality used, making operator bias unavoidable. Fourth, although the sample size was sufficient for descriptive comparison of SDR, the study may not have been adequately powered to detect differences in secondary outcomes such as ADR, advanced lesions, or adenoma miss rate. Finally, we did not assess interobserver variability or learning curves, which may influence SSL detection in real-world practice.

## 5. Conclusion

In conclusion, WAC combined with NBI was associated with a higher SSL detection rate than the comparator imaging strategies in this single-center retrospective cohort. Given the increasing recognition of SSLs as important precursors in the serrated pathway of colorectal carcinogenesis, these findings suggest that integrated imaging strategies may have clinical value in routine screening practice. However, causal inference cannot be made from this observational analysis, and future prospective multicenter studies are warranted to validate these results and evaluate cost-effectiveness and training requirements for implementation.

## Author contributions

**Conceptualization:** Bin Liu, Haiyan Wang, Lin Li, Xia Huang, Liangting Hou.

**Data curation:** Bin Liu, Haiyan Wang, Lin Li, Xia Huang, Liangting Hou.

**Formal analysis:** Bin Liu, Haiyan Wang, Lin Li, Xia Huang, Liangting Hou.

**Funding acquisition:** Bin Liu, Liangting Hou.

**Investigation:** Bin Liu, Liangting Hou.

**Writing – original draft:** Liangting Hou.

**Writing – review & editing:** Liangting Hou.
